# Household

**Published:** 1887-06

**Authors:** 


### HOUSEHOLD.

Coffee Ice Pudding.—Pound two ounces of fresh roasted coffee in a mortar, but do not reduce them to powder. Put them into a pint of milk with six ouuces of loaf sugar ; boil up, then leave it to get cold, strain it on to the yolks of six eggs in a double saucepan, and stir over the fire till the custard thickens. When quite cold, work into it one and a half gills of cream whipped to a froth, freeze it, lay it in a plain mould, and set in ice till wanted.

The Pipe Bread of Piedmont.—The dough is leavened and is formed of the finest wheaten flour, lightly salted, and kneaded into a stiff paste in a wooden bowl, which is covered and left for two hours to rise. Then it is rolled out and cut in pieces as thick and long as a stout finger. These pieces are laid side by side on a moistened paste-board. Let them stand to rise another hour ; then take up each piece by the two ends and gently draw it out about two or three feet in length. Arrange them on the oven shovel, which must be sprinkled with unsifted meal, and transfer them to the oven. A short time will bake them, when they must be cooled and kept in a dry place to become crisp. The secret of success with them is perseverance.

Simple Desserts.—Many housekeepers look upon all desserts in the light of luxuries, others, draw the line at dishes that call for eggs. Now, some dessert dish, if properly made, should form a part of every dinner, if fruit is not to be served. Even with fruit, some people require sugar. When no dessert is provided, a greater quantity of meat and vegetables must be eaten to satisfy the demands of Nature. For some this is all right, but for the majority of folk a certain amount of sugar and starch is necessary. Children should not be deprived of this kind of food. Even for the poor it is economical to provide a simple dessert. In arranging for a dinner, plan a light dessert when the rest of the meal is to be substantial. On the other hand, when the main part of the dinner is to be light, let the dessert be hot and substantial. For example, if the first part of the meal consists of cold meat and vegetables, or a hash and one vegetable, serve a hot apple pudding for dessert. A good one can be made of a pint of flour, prepared as for cream-of-tartar biscuit, rolled thin, and filled with pared and quartered apples, then steamed for two hours, and served with molasses or sugar sauce. Or, the apples may be put into a stewpan with a little water and sugar, or a little molasses, stewed for a few minutes, covered with the biscuit dough, and cooked for about ten minutes longer. No sauce will be needed with this pudding. Nothing could be cheaper, and it will be very palatable and wholesome. Apples may be added to boiled sago or tapioca, with a pleasing result. Soak a cupful of either tapioca or sago in three cupfuls of cold water over night ; then cook it in a double boiler for half an hour. Add to the contents of the boiler one cupful of sugar ; half a teaspoonful of salt, and two quarts of pared and quartered apples. Bake in a pudding dish for an hour and a quarter. Cool slightly, and serve with or without sugar and cream or milk. These puddings are so simple that they will not hurt even an invalid.—[*Maria Parloa, in Good Housekeeping.*

THE USES OF LEMONS.—Lemons are one of the most useful fruits in our domestic economy.

Lemonade is not only a luxury, but exceedingly wholesome. It is a good temperance drink.

The juice of half a lemon in a glass of water, without sugar, will frequently cure a sick headache.

If the hands be stained, there is nothing that will remove the stain better than a lemon or a lemon and salt.

After the juice has been squeezed from the lemon the refuse can be used for the purpose.

Lemon juice and sugar, mixed very thick, is useful to relieve coughs and sore throats. It must be very acid as well as sweet.

Lemon juice is also a very good remedy for rheumatism and the so-called biliousness of spring. In the latter case the juice should be taken before breakfast. The pulp may also be eaten, avoiding every particle of skin.

### THE MAID'S BEHEST.

FROM THE GERMAN OF ROBERT PRUTZ.

Moon, oh, moon! and didst thou see
How my lover kissed me?
Surely then I own to thee,
It hath sore distressed me.

But in sooth, I could not say
On my life to save it,
If *he* stole the kiss from *me*,
Or to *him* I gave it.

Thou must not the secret tell,
Darling moon, oh never;
If they ask thee what befell,
Be thou silent ever.

—*Translated for* HALL'S JOURNAL OF HEALTH.

